# Fracturing the affordance space: an account of digitalized alienation

**DOI:** 10.3389/fpsyt.2024.1407586

**Published:** 2024-09-03

**Authors:** Michael Butler

**Affiliations:** Department of Philosophy and Ethics, The University of North Dakota, Grand Forks, ND, United States

**Keywords:** alienation, digitalization, affordances, phenomenology, the self, embodiment, embodied cognition, ecological psychology

## Abstract

This paper investigates the lived experience of alienation as a form of mental strife or pathology as it is connected to the digitalization of modern life. To do so, I deploy the concept of affordances from ecological psychology, phenomenology, and embodied cognition. I propose an affordance-based model for understanding digitalized alienation. First, I argue that the lived sense of alienation is best understood as a fracturing of the affordance space, where possibilities for action are lived as disconnected from one another and therefore from one’s personal development and search for meaning. Using this model, I show how the process of digitalization can lead to a lived sense of alienation for modern subjects. On this model, digitalization is alienating insofar as it fractures the affordance space into disconnected fields that invite determinate, separate, and repeatable tasks—swiping, clicking, scrolling, etc.—rather than offering opportunities for the development of new cognitive and bodily skills that are mutually informing and enriching across different affordance fields.

## 1 Introduction

This paper investigates the lived experience of alienation as a form of mental strife or pathology as it is connected to the digitalization of modern life. Increasingly, the activities and interactions that make up our daily activities are mediated by digital devices and software. This includes not only the host of smartphone apps and wearable technology (like the Apple Watch) that inform our daily habits and behaviors but also processes that shape our broader social habits and ways of navigating spaces and gathering information, like digital kiosks at airports, restaurants, and museums; Learning Management Systems in educational contexts; QR codes on menus and advertisements; online platforms for shopping, dating, and socializing (i.e., Amazon, Tinder, and Twitter); and digitally mediated means of working remotely.

Digitalization is a decidedly modern phenomenon. For this reason, it makes sense that it would extend and engender the effects of modernity on human life and wellbeing. Since at least the 17th century, the rise of modernity has been accompanied by a lived sense of alienation that philosophers and social scientists have studied and remarked upon—Rousseau (1) arguably being the first and Hegel (2) and Marx (3) being perhaps the most famous. In the 20th century, alienation became the primary tool of analysis for social philosophy (4). While the accounts differ on the specifics, generally, alienation is understood as a social or psychological illness involving the problematic separation of subject and object that belong together (5). Beyond this broad and somewhat abstract definition, alienation is a slippery concept that has been used to describe a variety of such separations—alienation from one’s self or one’s consciousness (2), alienation from one’s environment, alienation from the body (6–8), alienation from the product or activity of labor (3), alienation from leisure (9), alienation from other people, etc. In addition to presenting as a modern problem of living in its own right, a sense of alienation has been associated with various conditions that may lead patients to seek out psychiatric treatment, such as depressed mood (10), anxiety symptoms (11), alcohol use disorders (12), psychological distress (13), insomnia (14), post-traumatic stress disorder (15), and suicidal ideation (16).

In this paper, I attempt to describe what is held in common within the various experiences of alienation beyond the rather abstract definition that Leopold (5) offers by deploying the concept of affordances from ecological psychology, phenomenology, and embodied cognition. I propose an affordance-based model for understanding digitalized alienation. I show how the lived sense of alienation is best understood as a fracturing of the affordance space, where possibilities for action are lived as disconnected from one another and therefore from one’s personal development and search for meaning. I demonstrate the viability of this model by applying it to two canonical cases of alienation—that of workers in 19th-century factories and that of people living with chronic illness. Finally, I show how this model is especially helpful for understanding the lived sense of alienation engendered by digitalization. I argue that digitalization is alienating insofar as it fractures the affordance space into disconnected fields that invite determinate, repeatable tasks—swiping, clicking, scrolling, etc. (17–19)—rather than offering opportunities for the development of cognitive and bodily skills that are mutually informing and enriching across different fields within one’s affordance space.

## 2 Embodiment, affordances, and sense of self

The purpose of this section is to introduce the concept of affordances as well as the related concepts of the affordance field and the landscape of affordances. I then advance a theory of self-development, drawing on Gallagher’s (20) idea of affordance space—a model for understanding a person’s life as a whole. On this model, as subjects grow and develop new skills, habits, and goals; they gain an increasingly complex sense of themselves and what is possible for them across diverse affordance fields. In doing so, they also develop a characteristic style of engagement with the world that is bodily and habitual—that is, they become who they are, unique from others.

The concept of affordances has its roots in existential and phenomenological philosophy. One of the fundamental insights of this tradition is that we do not live in our heads. Rather, we live in the world. That is to say that life goes on “out there” in front of us, where we encounter possibilities for action that are more and less likely to be engaged. Behind us lies the past—the places and scenarios we have come from and that have delivered us to the position we presently occupy. It is from this past, forgotten but preserved in our present orientation, that we approach the array of possibilities that lay before us.

Another way of putting this is to say that we exist in a situation—we find ourselves situated between a past of acquisition and a future that beckons us to take it up in terms of the habits, skills, and goals we have acquired.

This is evident through phenomenological reflection. Think for a moment about the experience of sitting down at a table with a cup of coffee to read a book. The world before you in such a scenario is not a blank slate or a collection of indifferent properties but a situation that calls for privileged modes of action and engagement based on a past of skill acquisition and habitual deployment. The cup of coffee, still too hot to sip, demands patience and promises the delayed possibility of pleasure. The chair that supports your body provides an orientation toward the novel you have brought along with you. It invites you to sink back into it and allows the novel to appear as a focal point. The novel itself bears the traces of your past in that you are part of the way through it. It falls open in your hands to the place you have marked and offers up the possibility of picking up where you left off, escaping into the world and problems of the characters and plot.

Such engagement in the world is bodily at the same time that it is cognitive. As phenomenologists like Husserl (21) and Merleau-Ponty (22) have noted, one’s intentional stance toward the world is typically lived as “I can” rather than as the Cartesian “I think”. While reading a book may be thought of as a purely cognitive activity, engaging with information that is contained in the symbolic representation of language on the page, it depends on a host of bodily skills and habits. Reading is made possible by a sort of default interpretation of the material that lies before you, carried out by your habitual disposition toward your objects (23). What allows the world to show up as a place to read depends on one’s bodily disposition toward these objects—the chair, the book, and the coffee can only appear for me as a situation that invites reading because I have learned to sit, to wait, to hold a book, and to grasp a cup. Likewise, my own idiosyncratic history of engagement with the world, through which I have developed the habits of a reader, is what allows the situation to appear as one in which I am at home. My book is marked on the page where I left off. The coffee, still too hot, provides a bridging continuity between my recent past—having just ordered it or made it—and my present and future.

For this reason, there is no universal situation. Our individual pasts carry forward an interpretive stance toward the world through our habitual embodied activity that renders an environment that solicits a response. We are not met with bare information to be processed by the software and hardware of our brains but with meaningfully weighted situations that invite us to take them up on the basis of our bodily past. For this reason, the same material configuration, for a different person, may invoke anxiety, stress, or boredom.

This situational nature of perceptual life is captured by the concept of affordances, first introduced by the psychologist James Gibson (24). Affordances refer to “what [the environment] offers the animal, what it provides or furnishes, either for good or ill” (24, 199). Put simply, subjects encounter possibilities for action and interaction in their environment, not determinate objects or collections of properties. That is, we do not directly perceive a white coffee cup but the possibility afforded to us by the coffee cup of grasping, drinking, throwing, avoiding, etc.

The idea is to draw our attention to the embodied and action-oriented nature of our pre-reflective perceptual life. We do not encounter a neutral or objective world but a world that speaks to us on the basis of the actions of which we are capable given the bodies we have. For me, a chair affords sitting and a novel affords reading because I possess a certain sort of body (flexible, mobile, joints, and limbs) and a certain set of skills (sitting and reading). It does not afford these possibilities to my dog, which has a much different bodily configuration and cannot read—though it may afford all sorts of other things to her. The appearance of the world understood through affordances is thus relative to an individual’s particular body and history. The world as I encounter it is relative to me, my body, and my skills. This is what is known as the field of affordances or “the affordances that stand out as relevant for a particular individual in a particular situation; i.e., the multiplicity of affordances that solicit the individual” (25).

The field of affordances can be broader than what is offered to an individual. I am also able to perceive not only what an environment affords for me but also what it may afford to someone else. Rietveld and Kiverstein (26) argued for what they call a “landscape of affordances”, wherein my lived environment does not only contain affordances relative to me and my idiosyncratic past but also what the environment affords to a “form of life” or a culture. If I live in a society where gambling is legal and common practice, I may perceive a casino or gaming table as affording the possibility of gambling even though I have never gambled, do not know the rules of any games, and am morally opposed to it. Likewise, someone who cannot read but who lives in a literate society can still encounter a book or a library as offering the affordance of reading to someone, but not me.

Thus, the field of affordances is also mediated by the culture in which our lives are embedded and our position within that culture. For example, in her discussion of the typical feminine bodily comportment, Iris Marion Young (27) showed how this general awareness of the affordances in one’s environment can be equally alienating—a rock face may be encountered as climbable for someone—but not for me, if I have learned to take up my body, as many young girls do in a sexist society, as something that may invite unwanted attention, may be fragile, and may need to be protected. For Young, this means that one’s experience of one’s body is lived as simultaneously immersed in and disconnected from one’s environment. The space surrounding a girl’s body, though it is within reach, does not invite action but rather invites one to withdraw. The body can be thus lived as an impediment to action. It can simultaneously be experienced as “I can” and “I cannot”.

The important thing here is that in the typical feminine bodily comportment that Young describes, a subject’s field of affordances is not informed only by the subject’s actual bodily capacities. Young notes that women and girls typically do not make use of the full range of motion available to them, despite the fact that they are able-bodied. Rather, the affordance space encountered is equally informed by social expectations of proper feminine behavior. This affects which affordances actually solicit action—actively calling out to a subject for engagement—and which are experienced as possibilities for someone else.

This points us to the concept of affordance space. Somewhere between the landscape of affordances available to a cultural form of life and the affordance field available in a given episode of perception (which may itself include a cultural awareness of affordances available to others) is what Gallagher (20) calls the affordance space. The affordance space is a topological model that is useful for understanding a person’s life as a whole. Gallagher (20) argued that our sense of self and what is possible for us develops throughout our lifetime along with the development of an individual affordance space or “the full range of possible affordance fields relative to an individual [ … ] including the current affordance field plus any possible changes in that field due to changes in physical or cognitive skills or environment”. As one’s skills develop, so too does one’s affordance space. Skills learned in one field may give rise to new affordances in new fields. Therefore, for instance, learning to write by hand with a pen and paper allows a whiteboard and marker to afford writing for a public audience. Taking that up and getting comfortable presenting ideas for an audience leads to new social affordances for ways of interacting with others in group settings. One encounters groups of others as affording the possibility of teaching, say. This in turn may transfer to more intimate relationships. One may take a more authoritative tone in one-on-one conversations and so on. In this way, we become who we are, that is, we develop a distinctive personal style, as we develop skills for coping with the world across different situations. In turn, as we encounter new situations, they offer affordances for engaging them with the particular set of skills we have developed. The world mirrors us back to ourselves, providing a theater for action and an environment for continued development. Thus, our sense of ourselves develops along with our awareness of our surroundings and what is or is not possible within them.

This happens both implicitly, as in the example above, and explicitly, in our own attempts to make sense of our life stories. A tension between different fields that calls our own self-conception into question may call for a re-evaluation and narration of who we consider ourselves to be (28). I may find myself solicited to be mean or impulsive in a particularly charged situation when I usually think of myself as a calm, collected, rational kind of person. Similarly, when we fail to meet the demands of a particular situation for which we thought we were well prepared or when we are making sense of a new situation (29, 30), we may be solicited to reflect upon ourselves and make sense of our lives and position within a task, culture, family, or profession. My sense of myself as a good teacher, say, may be called into question after a particularly difficult class where students seemed bored and the points I tried to make failed to land. Reflecting on such instances leads to the construction of a narrative sense of ourselves and our lives that may alter one’s approach to the world going forward and alter the shape of subsequent affordance fields one encounters as solicitous in one way or another (31).

What matters here is that one’s affordance space takes shape over time at the implicit level by the transfer of skills, strategies, goals, and habits developed in one context to another, and at the explicit level by soliciting subjects to make sense of the tension between various fields in a consistent narrative. Engaging the world this way, simply by moving through a diversity of situational affordance fields, gives rise to a personal style of habitual engagement across the affordance space—what we may call character or personality. We may be led to reflect on our style of engagement and self-consciously work on ourselves so as to develop a style of our own choosing or to challenge the sexist norms of society that have shaped our development. We also may not—but not being particularly prone to reflective thought or challenging problematic social norms is also an expression of our own personal style. The point is that who we are only emerges across affordance fields that demand new and different sorts of actions, behaviors, and self-evaluations, soliciting us to uniquely engage them on the basis of the habits, skills, and goals we have accrued in a way that expresses our bodily history of engaging the world.

## 3 Alienation

The purpose of this section is to demonstrate how alienation occurs when one’s affordance space becomes fractured. On the account I have been sketching out, we grow into ourselves at the same time that we learn to deal with an array of different affordance fields with a characteristic style that reflects our own past development back to us. To feel at home in the world is to feel well adapted to an affordance field. This is not to say that any case of maladaptation or not feeling at home constitutes a sense of alienation. The tension between multiple affordance fields or within a single affordance field can offer a productive opportunity for sense-making and self-development through which we develop new skills and new self-conceptions. To feel alienated, however, is to encounter an affordance field that does not address us as the particular people that we are but as anonymous and interchangeable with any other person. As noted at the outset, alienation is not a phenomenon that is reducible to something that is wrong with an individual. It arises as a result of social arrangements and relations that present individuals with alienating situations, addressing them on the basis of their membership in a pre-defined class or group with generic affordances on offer to anyone who belongs to that group rather than on the basis of their unique skills and capacities.

Marx famously described the situation of 19th-century factory workers this way. For Marx, one of the alienating characteristics of factory work is precisely how the field of possible actions is circumscribed for the worker as a worker. The work is dull and repetitive. It does not ask the worker to engage their mental faculties by problem-solving. Instead, it turns the worker’s body into an appendage of the machine. Again, there is little room for the development of new skills or the new application of old ones. The work demanded treats workers as interchangeable—indeed, this is the point, as it cheapens labor by doing so. On Marx’s account, to go to work is to step outside of the affordance field where one is at home and enter another one that is cut off and isolated from the rest of one’s life. When one is at work, one is not at home, and when one is at home, one is not at work (3).

Such alienation can also occur when one experiences one’s own body as alienating—as in chronic illness. Characteristic of such situations is the experience of one’s own body as an object to be maintained rather than as the transparent seat of consciousness from which one approaches a world of projects that matter to them. Such experience is not exclusive to chronic illness. From time to time, all of us experience our body this way—at the doctor’s office or the gym, for instance, where our bodies become something that we work on rather than with, or, as Sartre (7) describes, when we fall under the uninvited gaze of others. However, it is an experience that features prominently in first-person descriptions of living with chronic illness and phenomenological analyses thereof (cf. 6, 8). Jehangir Saleh, who died of cystic fibrosis in 2013, once described his own relationship with his body as such.

“I’ve mopped floors. Hopefully, all of you have, at some point, mopped the floor. Except, for me, the floor is my body. I get up in the morning, and inhale a bronchodilator. Then I inhale a mucolytic. This takes up about 35mins. Then, 30mins of postural drainage, followed by 10mins of breathing techniques. Then I inhale an anti-biotic, and then some anti-inflammatory medications: approx 2 hrs total. Then my day starts: I become a grad student. I write papers. Talk to students. Prepare for seminars. And then I come back home, and do this process all over again. All of this feels a bit like mopping a floor. The floor gets dirty, so you mop it up. The next day, the floor is dirty again: you mop it up. You’re never going to, once and for all, mop the floor. You’re never going to mop the floor in a way to end all floor mopping: someone is always going to be spilling something. And this is what it feels like, for me at least, do go through my medical routine: I get up in the morning. My lungs feel full of mucus. My airways feel tight. I get out the medical equipment, and I clean things up. And then I come home, and sure enough, my lungs feel full of mucus, and again, my airways feel tight. So I get out the medical equipment, and again, clean things up, again. This was aspect of CF life was emotionally difficult for me. Not because I don’t like domestic labor, but because for someone who spent his life being an over achiever, who felt like, with enough hard work, I could make things go my way, here was one thing that, no matter what I did, I couldn’t control.” (32)

In “mopping the floor” at the beginning and end of each day, Saleh’s body ceases to be the transparent envelope of his subjectivity, the orienting jumping-off point from which he moves toward the world—as it is in other situations. Instead, for him, the body is encountered as an imposition that demands labor that is not connected to the projects and actions that provide a sense of home—for Saleh, being a grad student and the typical activities connected to that. The body and its demands address him not as an idiosyncratic personality with a characteristic style but as a mechanistic caretaker, forced to maintain his body through the boring, alienating labor of inhaling medicine and performing repetitive physiotherapy.

On the account we are developing here, Saleh is describing the fracturing of his affordance space. The affordance space includes the various affordance fields available to him, but one—the field of “mopping the floor”—is lived as disconnected and cut off from others where he is able to move across a variety of different tasks with a personal style, in the process developing a more complex relationship to his books, ideas, peers, friends, and teachers. As a grad student, his situation calls on him to engage it in the style that has worked for him in the past. He feels “like an overachiever” who could “make things go his way”. Confronted by his body in illness, however, he is no longer afforded this possibility. His body, like the 19th-century factory, is always the same. It is always filling up with mucous, always requiring the same repetitive care. Nothing he does allows his situation to change, to reflect back to him the work he has done, to show progress, to see the effects of his actions. Instead, he has to step outside of himself for several hours each day and become his body’s anonymous caretaker.

From these two examples, we can draw out three prominent characteristics of alienating affordance fields. First, they are repetitive. They call for the same specialized but simple actions to be repeated. Second, they are unchanging. As with Saleh’s CF body and Marx’s workers, one is never finished with these tasks. They are there waiting for you at the start of the next day. No progress is ever made. There are no tangible or steadily improving results. At best, one can reconcile oneself to these actions, cope with them, and get through them. However, they are not going to become the scenes of one’s most meaningful, self-defining performances. Such fields fracture the continuity between the fields that make up the affordance space. By not offering the opportunity for the transfer of one’s skills, habits, and orientation, they hamper the development of a unique style of engagement across fields, producing fields that do not solicit subjects as unique individuals but as anonymous and interchangeable. They are affordance fields in which it is hard to feel at home because they do not solicit us in terms of our own making. Instead, they invite standardized, monotonous activity that does not lead to the development of new skills, goals, desires, or insights.

Third and finally, part of what makes these fields alienating is that the affordances on offer belong not to an individual with goals and desires that reflect his own sense of themselves but are on offer for a form of life. They are the sorts of affordances that are available to someone who belongs to a class or a culture, not a unique individual. In working at the factory or engaging in the work of “mopping the floor”, subjects are reduced to their identities as workers or CF patients. Jehangir Saleh, the promising grad student, is not who is solicited to inhale medicine and perform postural drainage. Importantly, this means that such tasks are lived as imposed from without by the facts of his body and the medico-therapeutic practices that have grown up around similar bodies. Likewise, the worker must go back to work each day, not because he wants to but because this is simply what one must do if one is a worker.

## 4 Digitalization and digitalized alienation

In this section, I offer an account of how digitalization engenders the lived experience of alienation. The point is not to claim that digitalization is only alienating. My point is more modest in two ways. First, alienation is only one of the possible effects of digitalization on human wellbeing.^1^ As a relatively new process in the modern era, but very much a modern process, it would make sense that digitalization would extend modernity’s effects on human wellbeing or minimally carry them forward—one of which is alienation. Working from this assumption, I sketch out a path for understanding digitalized alienation as a fractured affordance space. If, as I argued above, alienation can generally be thought of on this model, then using this model to analyze digitalized alienation makes sense. However, I make no claim about the priority or severity of alienation from digitalization’s other possible pernicious effects. Second, it is beyond the scope of this paper to offer a comprehensive account of all the ways that digitalization can be alienating. Rather, I aim to sketch out the structural components of digitalized alienation by thinking through a few prominent examples and abstracting a general account of digital alienation as the fracturing of the affordance space.

Digitalization is the process by which our everyday material environment becomes modulated and mediated by interactions with digital interfaces. This in turn shapes the affordances available to us in a given field. Perhaps the most obvious example of this is the prevalence of smartphones. As of 2021, more than 60% of the world population is using the internet, and most of these users do so by way of a smartphone. ^2^ Since its introduction in 2007, Apple’s iPhone and similar devices have replaced a plethora of more specialized bits and bobs we use to navigate urban life. In 2005, researchers working at Keio University and Intel Corporation’s People and Practices group conducted ethnographic research in London, Tokyo, and Los Angeles to identify patterns in the objects people carried in their pockets, wallets, and purses. What they found was a great deal of similarity in the sorts of things that city dwellers kept on their person despite being spread across three continents. Most common were photographs, icons and religious tokens, personal hygiene items, breath mints, keys, ID cards, transit passes, mobile phones, and money in various forms (36).

Almost 20 years later, many of these items have been replaced by the smartphone or, as the designers of our devices promise, soon will be. Apple and Google Pay can replace money, Instagram and a phone’s lock screen can replace photographic prints, and biometric identification and two-factor authentication can replace the need for ID cards and keys. Watches, clocks, datebooks, and address books, too, can be folded into the phone. Taking photos of parking spots or items we need on a grocery trip can replace note-taking. What matters here is that the activities we engage in carrying out the minutiae of our lives are increasingly afforded by a single object—a flat touchscreen that affords swiping and tapping. The countless engagements in the world that once required specialized equipment and unique movements—everything from unlocking the front door to getting on the bus or to showing off pictures of our loved ones—now require the use of a touchscreen (or soon could). Human interaction, too, is taking place more and more in the digital realm of the screen. McDonald’s, Chili’s, and similar chain restaurants have reduced their human staff by deploying touchscreen menu kiosks and mobile order and pay apps (37). Customer service and mental healthcare are increasingly carried out by Large Language Model chatbots accessed via instant messaging (38). Access to healthcare professionals, colleagues, and teachers is facilitated by video conferencing apps. Intimate relationships are maintained by sharing content across social media platforms and text messages.^3^

Along with this transformation of the material environment comes a transformation of the landscape of affordances available to the form of life that exists for contemporary subjects. There are norms of behavior, available to anyone with the minimum skills and embodiment, that shape what solicits our behavior. This becomes apparent in moments when we are otherwise idle—waiting in line, at a bus station, or restaurant—becomes a time to scroll Twitter or TikTok. Coffee shops are filled with laptops, tablets, and smartphones. The possibility of multiscreening—engaging more than one screen or window at a time while watching television or attending a virtual class—emerges. We set digital reminders and alarms to regulate our behavior and track our fitness progress with dedicated apps and wearable technology. The environment we grow into is increasingly one that solicits us to engage it via our connected devices. At the same time, this environment increasingly imposes these technologies upon us. One cannot go to school or work, hope to find a romantic partner, or make friends without access to the digital realm that such devices afford.

Such digitalization leads to a homogenization of our affordance fields. Simultaneously, this leads to the fracturing of our affordance space. As I argued above, affordance spaces develop through their diversity. When we are met with a variety of different affordance fields, we are given the opportunity for cross-field applications of new skills learned in different contexts. A style of engagement emerges across various fields, calling for different sorts of actions engaged in a similar style. When all situations begin to resemble one another in terms of what behaviors they invite and afford, we are met with fewer opportunities to develop a characteristic style, a way of being in the world that is uniquely our own. Less various affordance fields no longer afford the emergence of a consistent style across the affordance space. Thus, such a homogenization of affordance fields actually creates a fracturing of the affordance space where the utter sameness of the touchscreen interface requires less active adaptation of our skills across fields.

Digital devices fracture the affordance space by homogenizing affordance fields in two ways. First, they hamper the development of new skills and the deployment of old ones in new ways by reducing the number of tasks we perform simultaneously. What we think of as multitasking is actually a rapid switching between similar tasks rather than accomplishing two or more tasks at the same time. Second, they often do this by inviting or affording the same repetitive behaviors across a variety of unrelated affordance fields associated with different tasks. This fractures the affordance space by creating a metaphorical screen (often by deploying a material one) that separates one affordance field from others, preventing the possibility of cross-field adaptation and application of skills.

An example of the first is the use of automobile GPS navigation in the way it is presently configured. As Besmer (40) pointed out, GPS navigation systems privilege abstract space over lived places in their design and application. The resulting experience of using them separates two tasks usually associated with operating an automobile—driving and navigating (41). Driving involves operating the car, and navigating involves engaging one’s surroundings to find one’s way to one’s destination using environmental cues and affordances. By offloading most of the work involved in the navigation to the GPS, the duties of a GPS-aided driver are reduced to following the directions of a disembodied digital voice. This, likewise, reduces the operation of the car to its own more proximal task, not connected to the larger project of reaching one’s destination. Drivers concern themselves with the immediate affordance field and need not think about their broader position on the journey. This results in an affordance field that is cut off from one’s wider affordance space—including the place one is going and what one hopes to do there. The road in front of a driver simply affords to continue, perhaps by navigating the traffic immediately surrounding them. It does not necessarily present itself as a road *to anywhere*. As a result, one does not need to drive and navigate simultaneously in an affordance field that opens onto a wider affordance space and demands a subject adapt one’s driving and navigating skills to one another. One is no longer invited to become a skilled driver/navigator. Instead, the activity is reduced to engaging a single habitual skillset in a relatively proximal affordance field. Perhaps for this reason, drivers who use GPS are far less likely to remember how they got where they were going and take longer to learn their way around new places (42). Their driving activity is separated—and thus alienated—from their wider environments in this way.

The prevalence of smartphones creates a similar separation from one’s wider environment. The screens in our pockets provide a security blanket in that they are absolutely ready-to-hand (43) creating personal, proximal space within a wider public sphere (44, 45). I can retreat from the world in front of me into the screen and the comforting content it affords. However, the self that is solicited in such situations can resemble Saleh’s experience of mopping the floor. While the phone screen affords a retreat from a world that may seem uncertain or anxiety-inducing because it invites me to engage it in new ways, it does so by soliciting repetitive swiping and tapping. As Mark Kingwell notes, “The most significant feature [.] is not user, content, or platform, but instead the repeated finger flicks of the swipe”. Though we may seem more in control of our environments, in choosing to pull out our phones “the essence of the scene is the narrow way in which the user experiences himself or herself through the specific mechanism of this ‘choosing’” (17).

As with Marx’s factory workers, the activity supported by the phone does not culminate in a satisfying experience of completion. Whether one uses the phone to access audio, video, or textual content on social media, online shopping, dating, or working out “one can never come to the end of such experiences: there is always more being added to the feed [ … ] hence no opportunity for even the momentary sense of satisfaction” (17). The touchscreen offers the same affordance in all contexts. Every field affords the same activity. That activity never culminates. It never ends and opens onto a new field of affordances to be taken up in a new way. Thus, the affordances of such digital technologies are present across all fields. They invite us to step out of our wider surroundings and solicit us to re-enter an affordance field that treats us as anonymous interchangeable users engaging in repetitive behavior that does not yield a satisfying result. For users, this can be experienced as imposed as much as chosen. Like workers returning to the factory each morning, pulling out one’s phone in moments of idleness or using Google Maps to find their way to a new destination is simply what one does. Like Saleh attending to the needs of his body in illness, we count our steps and our reps at the gym and attend virtual therapy sessions to stave off the inevitable decay of our bodies and minds. We become our organism’s own anonymous caretakers.

## 5 Conclusion

The purpose of this paper was to demonstrate connections between the digitalization of modern life and the lived experience of alienation. To do so, I deployed the concept of affordances. In Section 2, I differentiated between the field of affordances (the episodic appearance of the world as affording action possibilities relative to an embodied subject and their skills, habits, and capacities) and the landscape of affordances (the possibilities on offer within a field relative to a form of life rather than an individual) and the affordance space (a topological model for understanding a person’s life as a whole including affordances that are available now or may become available in the future given a change in the subjects’ capacities or skills). Then, in Section 3, I showed how the lived experience of alienation can be thought of as a fracturing of the affordance space, where one or more affordance fields are lived as separate or cut off from others within an individual’s affordance space. The resulting experience is alienating insofar as one does not experience the world as affording the development of our affordance space by applying skills acquired elsewhere to new situations or developing new skills that can enrich our lives in other domains. I demonstrated the viability of this model by using it to analyze two canonical cases of alienation—that experienced by workers in 19th-century factories as described by Marx and that experienced by people with chronic illness as described by Saleh. Finally, in Section 4, I offer a theory for understanding how the process of digitalization, in transforming the landscape of affordances available to the modern form of life, can extend and engender the lived experience of alienation by creating environments and affordance fields that are cut off from other fields within one’s affordance space.

This is not to say that digital technology by virtue of its being digital must be necessarily alienating, nor is it to suggest that the experience of alienating affordance fields completely renders one’s entire life alienated and meaningless. The lived tension between an alienating affordance field and the rest of one’s life can lead to an opportunity for sense-making through reflection—as Saleh does in his own narrative reflection on his experience of living with CF or Young does in her phenomenological analysis of her own and other women’s observable hesitance in situations which demand goal-oriented physical activity. We can in this way seek to reappropriate our lives despite our fractured affordance spaces.

This is perhaps what is afforded by good talk therapy (20, 46) where the presence of a knowledgeable and empathetic clinician solicits a creative re-narration of one’s experience, affording the possibility of adopting a new, less alienated perspective on one’s own activity (23). As noted above, alienation is itself both a problem of living—something to be dealt with and overcome—and associated with a variety of other mental pathologies and strife that may lead a person to seek psychiatric treatment. Understanding alienation from the patients’ perspective as a fracturing of the affordance space could inform a therapeutic practitioner, providing insight into a client’s complaints. Therefore, the results of this study are potentially useful to counselors and psychiatrists in understanding the sources of patients’ complaints and mental strife and in designing and implementing therapeutic interventions.

## Author contributions

MB: Writing – review & editing, Writing – original draft.

## Footnotes

1 Others include loneliness (33), depression (11), addiction (34), and the degradation of ethical discourse (35).

2 Hootsuite. 2021. “The Global State of Digital 2021”. Online https://www.hootsuite.com/pages/digital-trends-2021. Thanks to Maciek Bednarski for this stat and source.

3 As of 2021, roughly seven in 10 Americans use some form of social media, a trend that remained relatively stable over the previous 5 years. Pew Research Center. (2021, April). Social Media Use in 2021 (39).
